# Molecular analysis of T^-^B^-^NK^+ ^severe combined immunodeficiency and Omenn syndrome cases in Saudi Arabia

**DOI:** 10.1186/1471-2350-10-116

**Published:** 2009-11-13

**Authors:** Osama Alsmadi, Abdulaziz Al-Ghonaium, Saleh Al-Muhsen, Rand Arnaout, Hasan Al-Dhekri, Bandar Al-Saud, Fadi Al-Kayal, Haya Al-Saud, Hamoud Al-Mousa

**Affiliations:** 1 Genetics Department, Research Center, King Faisal Specialist Hospital & Research Center, Riyadh, Saudi Arabia; 2 Department of Pediatrics, King Faisal Specialist Hospital & Research Center, Riyadh, Saudi Arabia; 3 Department of Pediatrics, College of Medicine, King Saud University, Riyadh, Saudi Arabia

## Abstract

**Background:**

Children with Severe Combined Immunodeficiency (SCID) lack autologous T lymphocytes and present with multiple infections early in infancy. Omenn syndrome is characterized by the sole emergence of oligoclonal auto-reactive T lymphocytes, resulting in erythroderma and enteropathy. Omenn syndrome (OS) shares the genetic aetiology of T^-^B^-^NK^+ ^SCID, with mutations in *RAG1, RAG2*, or *DCLRE1C*.

**Methods:**

Patients diagnosed with T^-^B^-^NK^+ ^SCID or phenotypes suggestive of Omenn syndrome were investigated by molecular genetic studies using gene tightly linked microsatellite markers followed by direct sequencing of the coding regions and splice sites of the respective candidate genes.

**Results:**

We report the molecular genetic basis of T^-^B^-^NK^+ ^SCID in 22 patients and of OS in seven patients all of Arab descent from Saudi Arabia. Among the SCID patients, six (from four families) displayed four homozygous missense mutations in RAG1 including V433M, R624H, R394W, and R559S. Another four patients (from three familes) showed 3 novel homozygous RAG2 mutations including K127X, S18X, and Q4X; all of which predict unique premature truncations of RAG2 protein. Among Omenn patients, four (from two families) have S401P and R396H mutations in RAG1, and a fifth patient has a novel I444M mutation in RAG2. Seven other patients (six SCID and one OS) showed a gross deletion in exons 1-3 in *DCLRE1C*. Altogether, mutations in *RAG1/2 *and *DCLRE1C *account for around 50% and 25%, respectively, in our study cohort, a proportion much higher than in previous reported series. Seven (24%) patients lack a known genetic aetiology, strongly suggesting that they carry mutations in novel genes associated with SCID and Omenn disorders that are yet to be discovered in the Saudi population.

**Conclusion:**

Mutation-free patients who lack a known genetic aetiology are likely to carry mutations in the regulatory elements in the SCID-causing genes or in novel genes that are yet to be discovered. Our efforts are underway to investigate this possibility by applying the whole genome scans on these cases via the use of Affymetrix high density DNA SNP chips in addition to homozygosity mapping.

## Background

Severe Combined Immunodeficiency (SCID) is characterized by a block in T lymphocyte differentiation that is variably associated with abnormal development of other lymphocyte lineages, i.e. B or NK lymphocytes or more rarely of the myeloid lineage [1,2]. The overall SCID frequency is estimated to be 1 in 75,000-100,000 of live births [3,4]. The clinical presentation is fairly uniform and is characterized by early onset and diverse infections. Oral candidiasis, persistent diarrhea with growth impairment and/or interstitial pneumonitis are the most frequent infectious manifestations leading to diagnosis [5].

SCID is charecterized by high level of genetic and clinical heterogeniety, as more than 10 conditions have been identified and can be distinguished according to cellular phenotype, inheritance pattern, and the responsible genes [6-12]. Infants with autosomal recessive SCID caused by mutations in recombination activating genes 1&2 (*RAG1 *&*RAG2*) [13] that are necessary for the somatic rearrangement of antigen receptor genes on T- and B-lymphocytes [14,15], or in *DCLRE1C *(*Artemis*) [16], resemble all other forms of SCID in their infection susceptibility, however their lymphocyte phenotype is charecterized by predominantly circulating NK cells and undetectable B or T lymphocytes (T^-^B^-^NK^+ ^SCID) [13]. *RAG1*, *RAG2*, and *DCLRE1C *are the primary genes responsible for the T^-^B^-^NK^+ ^SCID phenotype [17] and in a recent report, mutations in *LIG4 *were also documented in patients with this phenotype who also have microcephaly and developmental delay [18].

In addition to causing the SCID phenotype, hypomorphic mutations in *RAG1 *or *RAG2 *can lead to partially impaired V(D)J recombinational activity resulting in Omenn syndrome (OS) [19,20]. OS can also result from defects in other genes including *DCLRE1C *[21], *LIG4 *[22], IL7RA [23], common gamma chain [24], ADA [25], RMRP [26] and CHD7 [27]. In OS, the absolute lymphocyte count is elevated due to circulating non-functional oligoclonal T lymphocytes [28,29]. There is also a third group of patients, called "atypical SCID/OS" or "leaky SCID" patients because the clinical features do not exactly match those of the previous two categories of patients [5].

A high number of patients bearing mutations in RAG genes has been reported so far (RAGbases are freely accessible on the web at http://www.uta.fi/imt/bioinfo/RAG1base and http://www.uta.fi/imt/bioinfo/RAG2base). Various mutations have been identified, both in *RAG1 *and *RAG2*, which can be either severe, leading to null alleles, or mild, leading to hypomorphic alleles that can still maintain a residual enzymatic activity. Null mutants typically predominate in classical T^-^B^- ^SCID, as no productive rearrangement of the T cell receptor (TCR) or B-cell receptor (BCR) can occur, while missense mutations predominate in OS and leaky SCID [30].

The same mutation in different individuals usually lead to similar phenotype. There are also a few cases in which the same mutation gives rise to different clinical presentation [5,19] suggesting that epistasis or other as yet unknown factors may play a role in determining the clinical picture and outcome.

Little is known about the molecular aspects of SCID or Omenn syndrome in Saudi patients. The incidene of SCID in Saudi population (representative of Arabian populations) is not well established but an initial data have suggested a 20 fold increase relative to the international figures [31]. This is mostly due to the high rate of consanguinity in the country (56%) [32]. Similar finding can be also extrapolated from the Iranian registery for primary immune deficiencies [33]. The underlying molecular genetic defects responsible for SCID and OS in Saudi population have not been previously studied. In this report, we document for the first time the molecular findings on Saudi patients with T^-^B^-^NK^+ ^SCID and Omenn syndrome.

## Methods

### Patients

On the basis of the clinical presentation and the immunologic data the patients were divided into 2 subgroups, T^-^B^-^NK^+ ^SCID and Omenn syndrome. Total of 22 patients with T^-^B^-^NK^+ ^SCID phenotype and seven Omenn syndrome patients who have been followed by the immunodeficincy clinics at King Faisal Specialist Hospital and Research Center (KFSHRC) were enrolled in this study. The clinical and immunological characterestics of all patients are shown in Additional Files 1 &2. All patients were screened for mutations in *RAG1, RAG2*, and *DCLRE1C *genes. Patients who are negative for all three genes were then sequenced for *LIG4*. This study was approved by the Institution Review Board (IRB) at KFSHRC, and an informed consent was obtained for each of the participating patients.

### Cellular and immunological assays

Peripheral leukocyte markers were determined with immunofluorescence staining and flow cytometry [34] using labeled antibodies for T cells (CD3, CD4 and CD8), natural killer cells (CD16 and CD56), and B cells (CD19) (Antibodies were acquired from Becton, Dickinson & Co, San Jose, California, USA). T-cell function was determined in vitro by proliferative responsiveness to phytohemagglutinin stimulation as described [35]. Serum Ig levels were measured by nephelometry [36].

### Clinical samples and DNA isolation

For prospective patients, peripheral blood samples were obtained from the patients and parents through venipuncture. Genomic DNA was then extracted from the blood samples using standard techniques [37]. For patient who underwent stem cell transplantion before the study initiation, pretransplant stored DNA was obtained from the HLA typing laboratory at KFSH&RC for molecular analysis.

### Genotyping by microsatellite markers

Homozygosity mapping based on the utilization of microsatellite markers flanking *RAG1/RAG2 *(D11S4203, D11S4083, and D11S4102) or *DCLRE1C *(D10S1725, D10S191, and D10S1653) was used as a prerequisite for the whole candidate gene sequencing. Inversely, heterozygosity was used for exclusion of whole gene sequencing. The rationale for using homozygosity mapping was because the studied cases in this report were almost exclusively from consanguineous families (patient 19 in Additional File 1 was the only exception), and hence an autosomal recessive homozygous founder mutation is likely to be the cause of the observed phenotype in these cases. For *LIG4*, direct sequencing of the coding region of this gene was implemented without the microsatellite evaluation; this gene is constituted by 2 exons and only the second exon is coding. For genotyping, PCR amplification was performed on a thermocycler (DNA Engine Tetrad. MJ Research, USA) in a total volume of 25 μl, containing 10 ng DNA, 50 mM KCl, 10 mM Tris-HCl (pH 9.0), 1.5 mM MgCl2, 0.1% Triton X-100, 0.25 mM of each dNTP, 0.5 pM of each primer (one fluorescently labeled), and 1 Unit of Taq polymerase (QIAGEN, D-40724, Hilden, Germany). Following the assembly of the reaction mix, PCR was carried out by an initial denaturation step at 95°C for 15 min followed by 30 cycles of denaturation at 95°C for 30 sec, annealing at 50°C for 30 sec and extension at 72°C for 30 sec followed by a final extension step at 72°C for 4 min. Amplification products were separated using a MegaBace 1000 capillary sequencer and sized using the Genetic Profiler software package (Amersham, Sunnyvale, CA, USA).

### Sequencing of *RAG1*, *RAG2*, *DCLRE1C*, and *LIG4*

Coding sequences of *RAG1, RAG2, DCLRE1C*, and *LIG4 *were amplified from genomic DNA. DNA samples were obtained before stem cell transplantation of the participating patients. DNA was also obtained from the parents when possible. Prior to a full gene sequencing, genotyping of three markers flanking the above genes was usesd as a gene exclusion criterion when heterozygosity is established. Sequencing primers were designed for the amplification of the four genes based on the sequences reported in databases (*RAG1*, M29474; *RAG2*, M94633; *DCLRE1C*, NM_022487; *LIG4*, NM_0002312). Sequencing primers were designed and optimized for the entire coding region of each of the three genes. Primer sequences and PCR conditions are available from the authors upon request. PCR amplifications were performed in 25 μl reactions as previously described [38]. PCR products were sequenced directly using the DYEnamic ET Dye Terminator Cycle Sequencing Kit (Amersham Biosciences; Piscataway, NJ, USA; http://www.amersham.com) on a MegaBACE 1000 DNA Analysis System (Molecular Dynamics; Sunnyvale, CA, USA). Sequence data were aligned against the reference GenBank sequences and examined for variation. For novel mutation verification, anonymized 96 DNA samples derived from normal Saudi blood donors were sequenced.

## Results

### Patient clinical and immunological characteristics

#### T^-^B^-^NK^+ ^SCID phenotype

Twenty two patients belong to this category were identified and recruited. They presented with the typical clinical manifestations including chronic diarrhea, failure to thrive, severe opportunistic infections, lymphopenia, absent or reducded T- and B-lymphocytes, hypogammaglobulinemia, and poor lympohocytes response to mitogen stimulation (Additional File 1).

#### Omenn phenotype

Seven patients were identified with the typical clinical presentation of diffuse exfoliative erythroderma, chronic diarrhea, failure to thrive, severe opportunistic infections, generalized lymphadenopathy, and hepatosplenomegaly. They had detectable activated T-lymphocytes with low circulating B-lymphocytes and no evidence of maternal T-cell engrafment as indicated by the short tandem repeat (STR) analysis, hypogammaglobulinemia, and poor lympohocytes response to mitogen stimulation (Additional File 2). None of our patients had microcephaly or severe developmental delay.

The overwhelming majority of these patients 28/29 (97%) belong to consanguineous parents all of which were of Saudi decent. None of our patients had a family history typical of X-linked inheritance although this could not be definitely ruled out in other Saudi families where the affected individuals are only males.

### Genotyping of OS and T^-^B^-^NK^+ ^SCID patients

RAG1/2 locus genotyping was performed by using a set of three microsatellite markers (D11S4203, D11S4083, and D11S4102) spaning a ~1 cM interval on chr 11. A second set ot 3 microsatellite markers (D10S1725, D10S191, and D10S1653) spanning a 2 cM locus on chr 10 which harbors *DCLRE1C *was also used. 22 patients with the T^-^B^-^NK^+ ^SCID phenotype and seven patients with OS were genotyped by using both marker sets. A gene locus was considered homozygous if at least the second (central) marker in each marker set is homozygous; in such instances the respected gene's whole coding regions were subsequently analyzed for mutation by direct sequencing. From the overall 29 patients, homozygosity was demonstrated in 19 for the *RAG1/2 *locus, and in seven for the *DCLRE1C *locus. Homozygous *RAG1 *or *RAG2 *mutations were detected (as will be described in the next section) only in 15 leaving four without a detectable mutation (mutation-free). One or both parents of these four mutation-free patients were curiously homozygous for the *RAG1/2 *locus. All seven patients homozygous for the *DCLRE1C *locus subsequently showed a novel gross deletion in *DCLRE1C*.

### Gene sequencing for T^-^B^-^NK^+ ^SCID and OS patients

Twenty two patients (P1-P22) (Additional File 1) with T^-^B^-^NK^+ ^were identified and screened for mutation in *RAG1*, *RAG2*, and *DCLRE1C*. Patients who did not show presence of mutation in any of these three genes were subsequently screened for mutation in *LIG4 *by direct gene sequencing. Ten patients were positively identified with homozygous mutations in RAG1/2 (Additional File 3). Among them, six (from four families) have four different missense mutations in *RAG1*. The remaining four positive patients (from three familes) have three novel homozygous *RAG2 *nonsense mutations.

Parents of all ten patients with *RAG1 *or *RAG2 *mutations were confirmned as carriers (heterozygous) of the respective mutation. Six patients showed a novel gross deletion mutation spanning exons 1-3 in *DCLRE1C*. No mutation was detected in the coding regions of *RAG1*, *RAG2 *or *DCLRE1C *genes for any of the remaining 6 T^-^B^-^NK^+ ^SCID patients (P16-P21; ~27%).

Five families with Omenn syndrome (OS) that include seven affected patients (OS1-OS7) were also studied (Additional File 2). Variable homozygous mutations, including one novel, were identified in all families except one (F2) (Additional File 4); no mutation in *RAG1*, *RAG2*, or *DCLRE1C *was found in OS3 (F2) patient. OS7 (F5) patient showed the presence of the three-exon deletion in *DCLRE1C *that was also seen previously in the T^-^B^-^NK^+ ^SCID patients. Parents of the patients with positive *RAG1 *and *RAG2 *mutations were confirmned as carriers of the respective mutation.

## Discussion

In this communication, we are reporting the molecular characterization of a cohort of 29 Saudi patients, 22 of which are of the T^-^B^-^NK^+ ^SCID phenotype and seven with Omenn syndrome. Although the underlying molecular causes of T^-^B^-^NK^+ ^SCID and Omenn syndrome are well established world-wide, the aetiology of these disorders is yet to be defined in Saudi population.

Within the 22 T^-^B^-^NK^+ ^SCID patients, ten (46%) were found to carry homozygous mutations in *RAG1 *or *RAG2*, and six (27%) in *DCLRE1C*. The remaining six patients (27%) had no detectable mutation in the coding regions of *RAG1*, *RAG2*, or *DCLRE1C*. None of those patients were tested for radiosensitivity to exclude the recently described causes of T-B-NK+ SCID such as Cernunnos [39] or DNA-PK deficiency [40]; the lack of growth retardation and microcephaly, however, argues against Cernunnos deficiency but does not exclusively rule out the involvement of either of these 2 genes.

Out of 6 *RAG1 *mutations identified, four proviously reported (R396H, R394W, S401P and V433M), are interestingly localized within the nonamer binding domain (NBD) which is constituted of a 56 amino acid strech between residues 392-447 in the encoded 1040-aa RAG1 protein http://www.expasy.org/uniprot/P15918. R394W and R396H are critically present within the highly-conserved N-terminus amino acids 392-396 of the NBD which is composed of 5 unique residues (^392^G^393^G^394^R^395^P^396^R). The resulting non-conserved amino acid substitutions within this conserved domain will most likely result in profound loss of function of RAG1, and hence abrogation of the protein's interaction with the recombination signal sequence (RSS) during cellular DNA recombination. This notion is supported by the severly reduced number of circulating T- and B-lymphocytes in these patients combined with a severe reduction in Ig levels (Additional Files 1 &2). Furthermore, previous reports also suggested mutations of the basic residues in this conserved domain had led to complete loss of RAG1 function as demonstrated by inability of the NBD-mutated RAG1 to mediate V(D)J recombination [41-43]. The fifth *RAG1 *mutation, R559S, on the other hand is localized to the catalytic domain of RAG1 which interact with RAG2. R559S is a destructive non-conservative change from large size and basic (R) to small size and polar (S) amino acid, a change that is likely to disturb the conformational structure of RAG1 as a consequence, and hence abrogation of its ability to mediate V(D)J recombination. This speculation is indeed supported by a previous report describing the same mutation (R559S) to be associated with a reduced ability to mediate V(D)J recombination [44]. Similar to R559S, R624H is also a non-conservative change from large size and basic (R) to medium size and polar (H) amino acid. This change is also likely to disturb the structure of RAG1 because of the mutation's critical location in the RAG2-interacting core region. R624H among other mutations located in the core region of RAG1 were shown to result in a dramatic reduction in the mutant RAG1 recombination frequencies compared to the wild-type RAG1 [19].

RAG2 mutations (Q4X, S18X, and K127X) found in the T^-^B^-^NK^+ ^SCID patients were very intreguing since all of them were of the early truncation type. Even if the mutatnt transcripts escape the nonsense-mediated mRNA decay (NMD), the protein they encode would be missing the key RAG2 domains, including the active core within which lies the nuclear localization signal (NLS). Therefore, the associated phenotype in the patients with these truncation mutations is most probably a reflection of a failed RAG1-RAG2 interaction which occurs normally in the nucleus, and is required during T-cell receptor (TCR) and B-cell immunoglobulin (Ig) rearrangements [5,28]. The fourth RAG2 missense mutation, I444M, was detected in one patient (OS6). As shown in Additional File 2, this patient has only a marginal total lymphocyte count (620/mm3), and thus the patient may have a residual RAG2 activity. This is an appealing proposal because both I&M amino acids are of similar physico-chemical properties, and both are medium size and hydrophobic. This mutation is probably of the hypomorphic type which is consistant with the mild phenotype seen in this patient.

Previous reports have revealed that truncated core proteins, encompassing amino acids 384-1008 for RAG1 and amino acids 1-387 for RAG2, are necessary and sufficient to rearrange artificial V(D)J recombination substrates in vitro [45,46]. Our study has identified 8 homozygous missense and 3 truncation mutations (Additional Files 3 &4) that all map to the RAG1 and RAG2 core regions. In case of the *DCLRE1C *three-exon deletion (exons 1-3) that we identified in seven affected patients (six T^-^B^-^NK^+ ^SCID and one OS), it is anticipated that such mutaion will lead to a non-functional truncated protein product (provided the intact exons 4-14 are transcribed and translated). Unfortuanately, further molecular investigation is not possible because this is a retrospective study and these patients had already received bone marrow transplantation. Interestingly however is that this mutation is capable of producing either SCID or OS phenotypes, as seen in our patients.

In correlating genotype to phenotype, it is clear that all RAG1 and RAG2 mutations reported here, with the exception of I444M in OS2, are also associated with severe SCID or OS. Additionally, it is also noteworthy that all our patients who are negative for mutations in *RAG1, RAG2*, or *DCLRE1C *were also negative for *LIG4 *indicating that mutations in the latter are not a common cause of SCID or OS in Saudi patients. This is consistant with data reported previously in other populations [5]. Genotyping and sequencing results for cases in which multiple tribe-specific *RAG1/2 *mutations were detected excluded the common founder hypothesis for this subset of patients; however the *DCLRE1C *3-exon deletion detected in several other patients form different families may either be a recurrent or a founder one and remains open for further exploration.

## Conclusion

This study shows that mutations in *RAG1/2 *and *DCLRE1C *are seen in different Saudi patients with SCID or OS phenotype. Given the observed level of consanguinity which approaches 100% we believe that genomewide homozygosity mapping is likely to reveal novel loci associated with these phenotypes in the mutation-free patients for whom linkage to the above 3 loci has been excluded and this is being actively pursued by our group.

## Competing interests

The authors declare that they have no competing interests.

## Authors' contributions

OA wrote the manuscript, designed the study, and conducted the genetic analysis and interpretation. AG, SM, RA, HD and BS carried out the patient clinical evaluation. FA and HS carried out the genetic and sequence analyis. HM co-designed the study, wrote the clinical summary of the patients, and supervised the clinical evaluation and patient chart review. All authors read and contributed to the manuscript writing.

## Pre-publication history

The pre-publication history for this paper can be accessed here:

http://www.biomedcentral.com/1471-2350/10/116/prepub

## Supplementary Material

Additional file 1 **Table S1 - Laboratory and clinical characteristics of patients with T^-^B^-^NK^+ ^severe combined immunodeficiency**. Laboratory and clinical characteristics. Click here for file

Additional file 2 **Table S2 - Laboratory and clinical characteristics of patients with Omenn Syndrome**. Laboratory and clinical characteristics. Click here for file

Additional file 3 **Table S3 - Mutations detected in patients with T^-^B^-^NK^+ ^Severe combined immunodeficiency**. Mutations listing. Click here for file

Additional file 4 **Table S4 - Mutations detected in patients with Omenn syndrome**. Mutations listing. Click here for file
