# Supplementary material for: Molecular analysis of T-B-NK+ severe combined immunodeficiency and Omenn syndrome cases in Saudi Arabia
Source: BMC Med Genet. 2009 Nov 13;10:116. doi: 10.1186/1471-2350-10-116 (PMC2780402; doi:10.1186/1471-2350-10-116)
Supplement: Additional file 1 — Table S1 - Laboratory and clinical characteristics of patients with T-B-NK+ severe combined immunodeficiency. Laboratory and clinical characteristics. [file 1471-2350-10-116-S1.doc]

# Table 1- Laboratory and clinical characteristics of patients with T^-^B^-^NK^+^ Severe combined immunodeficiency

| Patients | Age  at diagnosis  (months) | Lymph-ocytes /mm^3^ | CD3  /mm^3^  (%) | CD4  /mm^3^  (%) | CD8  /mm^3^  (%) | CD19  /mm^3^  (%) | CD56/16  /mm^3^  (%) | IgG g/L | IgA g/L | IgM g/L | IgE  Ku/L | PHA  CPM | Clinical Presentation |
| --- | --- | --- | --- | --- | --- | --- | --- | --- | --- | --- | --- | --- | --- |
| 1 | 4 | 2708 | 108 (4) | 135  (5) | <27  (<1) | <27  (<1) | 2546  (94) | <1.4 | <0.25 | 0.31 | <2 | 1011 | Chronic diarrhea, F.T.T., BCGitis, Recurrent chest infection, S/P, BMT |
| 2 | 1 | 308 | <3  (<1) | <3  (<1) | <3  (<1) | 6  (2) | 283  (92) | 9.1 | <0.25 | <0.17 | ND | 288 | Newborn screening, S/P BMT |
| 3 | 7 | 418 | <4  (<1) | <4  (<1) | <4  (<1) | <4  (<1) | 405  (97) | 1.7 | <0.25 | <0.18 | ND | 214 | Dessiminated BCGitis, CMV infection, Chronic diarrhea, F.T.T., Recurrent chest infection, S/P, BMT |
| 4 | 3 | 172 | <2  (<1) | <2  (<1) | <2  (<1) | <2  (<1) | 153  (89) | 1.9 | <0.25 | <0.18 | ND | 1138 | Chronic diarrhea, CMV infection, Chest infection |
| 5 | 4 | 516 | <5  (<1) | <5  (<1) | <5  (<1) | <5  (<1) | 489  (95) | 1 | <0.3 | <0.2 | ND | 217 | Recurrent chest infection, F.T.T., S/P BMT |
| 6 | 1 | 659 | <7  (<1) | <7  (<1) | <7  (<1) | <7  (<1) | 638  (97) | 8.9 | <0.25 | <0.17 | <2 | 221 | Newborn screening, S/P BMT |
| 7 | 5 | 810 | <8  (<1) | <8  (<1) | <8  (<1) | <8  (<1) | 760  (95) | <1.8 | <0.07 | <0.04 | <5 | ND | Chronic diarrhea, Oral thrush, Family history of 2 death, S/P BMT |
| 8 | 1 | 978 | <10  (<1) | <10  (<1) | <10  (<1) | <10  (<1) | 918  (93) | ND | ND | ND | ND | 118 | Newborn screening, Chronic diarrhea, S/P BMT |
| 9 | 5 | 489 | <5  (<1) | <5  (<1) | <5  (<1) | <5  (<1) | 453  (94) | 1.3 | <0.25 | <0.17 | <2 | ND | Chronic diarrhea, F.T.T., Dessiminated BCGitis, S/P BMT |
| 10 | 6 | 167 | 2  (1) | <2  (<1) | <2  (<1) | 2  (1) | 165  (99) | 4.2 | <0.25 | <0.18 | <2 | 3175 | Chronic diarrhea, FTT, recurrent chest infections, CMV infection, s/p BMT |
| 11 | 4 | 417 | <4  (<1) | <4  (<1) | <4  (<1) | <4  (<1) | 370  (89) | 1.5 | <0.3 | <0.2 | 29 | ND | Fever, stridor, Oral thrush, Recurrent infection, S/P, BMT |
| 12 | 1 | 256 | <3  (<1) | <3  (<1) | <3  (<1) | <3  (<1) | 219  (85) | 4.4 | <0.3 | <0.2 | ND | ND | Newborn screening, Preterm 29 wks, S/P BMT |
| 13 | 4 | 75 | <1  (<1) | <1  (<1) | <1  (<1) | <1  (<1) | 69  (92) | <1.4 | <0.25 | <0.17 | <2 | ND | Dessiminated BCGitis, F.T.T., Chronic diarrhea, S/P BMT |
| 14 | 1 | 1008 | <10  (<1) | <10  (<1) | <10  (<1) | <10  (<1) | 877  (87) | 12.1 | <0.25 | <0.17 | ND | 996 | Newborn screening, S/P BMT |
| 15 | 1 | 1034 | <10  (<1) | <10  (<1) | <10  (<1) | <10  (<1) | 993  (96) | 4.8 | <0.25 | <0.18 | ND | 511 | Newborn screening, Chronic diarrhea, S/P BMT |
| 16 | 12 | 2660 | 158  (6) | 106  (4) | 26  (1) | 26  (1) | 2218  (84) | 3.2 | <0.23 | 1.54 | 39 | 487 | Recurrent chest infection & OM, Oral thrush, F.T.T., Chronic diarrhea, BCGitis, S/P BMT |
| 17 | 14 | 1258 | 226  (18) | 138  (11) | 13  (1) | 50  (4) | 931  (74) | 5.4 | 3.09 | 3.41 | <2 | 1411 | Recurrent chest infection, Chronic diarrhea, F.T.T., S/P BMT |
| 18 | 5 | 928 | <9  (<1) | <9  (<1) | <9  (<1) | <9  (<1) | 863  (93) | <1.4 | <0.25 | <0.18 | <2 | ND | Disseminated BCGitis, Chronic diarrhea, F.T.T., Retropharyngeal abscess, S/P BMT |
| 19 | 2 | 265 | 125  (47) | 93  (35) | 13  (5) | 53  (20) | 101  (38) | 5.1 | <0.25 | 0.39 | 24 | 2785 | Chronic diarrhea, F.T.T., S/P BMT |
| 20 | 8 | 211 | <2  (<1) | <2  (<1) | <2  (<1) | <2  (<1) | 175  (85) | 1.8 | <0.25 | <0.17 | ND | ND | Chronic diarrhea, Recurrent chest infection, S/P, BMT |
| 21 | 1 | 98 | <1  (<1) | <1  (<1) | <1  (<1) | <1  (<1) | 86  (86) | 10.1 | <0.25 | <0.17 | <2 | ND | Newborn screening, S/P BMT |
| 22 | 2 | 576 | <6  (<1) | <6  (<1) | <6  (<1) | 17  (3) | 437  (76) | 1.6 | <0.23 | <0.17 | <2 | 610 | Recurrent chest infection, BCGitis, S/P BMT |

Note:

Normal reference values: Lymphocytes 4000-12000 per mm^3^, CD3 3100-4800 per mm^3^, CD4 2200-3300 per mm^3^, CD8 1100-1700 per mm^3^, CD19 1100-1900
per mm^3^, CD16^+^56^+^ 300-700 per mm^3^, IgG 2.5-9.1 g/L, IgA 0.2-1.2 g/L, IgM 0.2-1.5 g/L, IgE 1.6-30 g/L and PHA 94935-171149 CPM.

FTT: failure to thrive

s/p BMT: status post Bone Marrow Transplantation

CMV: cytomegalovirus

OM: otitis media

ND: not done
