# Supplementary material for: Molecular analysis of T-B-NK+ severe combined immunodeficiency and Omenn syndrome cases in Saudi Arabia
Source: BMC Med Genet. 2009 Nov 13;10:116. doi: 10.1186/1471-2350-10-116 (PMC2780402; doi:10.1186/1471-2350-10-116)
Supplement: Additional file 2 — Table S2 - Laboratory and clinical characteristics of patients with Omenn Syndrome. Laboratory and clinical characteristics. [file 1471-2350-10-116-S2.doc]

**Table 2- Laboratory and clinical characteristics of patients with Omenn Syndrome**

| Patients | Age at diagnosis (months) | Lymph-ocytes /mm^3^ | CD3  /mm^3^ (%) | CD4  /mm^3^ (%) | CD8 /mm^3^ (%) | CD19 /mm^3^  (%) | CD56/16 /mm^3^  (%) | DR (%) | IgG g/L | IgA g/L | IgM g/L | IgE  Ku/L | Mat. Cell | PHA  C.P.M. | Clinical Presentation |
| --- | --- | --- | --- | --- | --- | --- | --- | --- | --- | --- | --- | --- | --- | --- | --- |
| OS1 | 3 | 2156 | 496  (23) | 431  (20) | 86  (4) | 22  (1) | 1595  (74) | 16 | <0.29 | <0.04 | <0.02 | <2 | - ve | 2362 | Chronic diarrhea, F.T.T., Exfoliative dermatitis, S/P BMT |
| OS2 | 1 | 976 | 605  (62) | 605  (62) | 10  (1) | <10  (<1) | 332  (34) | 67 | 5.9 | <0.25 | <0.17 | ND | - ve | 1064 | Chronic diarrhea, F.T.T., Exfoliative dermatitis, S/P BMT |
| OS3 | 3 | 5850 | 5441  (93) | 1872  (32) | 3393  (58) | 59  (<1) | 293  (37) | 62 | 3.3 | <0.25 | <0.17 | ND | - ve | 1802 | Newborn screening, Chronic diarrhea, Exfoliative dermatitis, S/P BMT |
| OS4 | 1 | 2156 | 1100  (51) | 1035  (48) | 43  (2) | <22  (<1) | 798  (37) | 62 | 3.3 | <0.25 | <0.17 | ND | - ve | 1802 | Newborn screening, Chronic diarrhea, Exfoliative dermatitis, S/P BMT |
| OS5 | 1 | 699 | 336  (48) | 245  (35) | 119  (17) | <7  (<1) | 266  (38) | 24 | 4.2 | <0.25 | 0.28 | ND | - ve | 2772 | Severe dermatitis, Chest infection, Sepsis, Chronic diarrhea, CMV, Candidemia, S/P BMT |
| OS6 | 3 | 620 | 415  (67) | 347  (56) | 25  (4) | <6  (<1) | 180  (29) | 29 | 1.2 | <0.25 | <0.18 | 3.1 | - ve | 14625 | Recurrent chest infection, Chronic diarrhea, F.T.T., eczema, BCGitis, CMV infection, Died of sepsis |
| OS7 | 8 | 7800 | 5694  (73) | 5226  (67) | 390  (5) | <78  (<1) | 1716  (22) | 71 | 1.2 | <0.25 | 0.17 | <2 | - ve | 2978 | Recurrent chest infection, Chronic diarrhea, exfoliative dermatitis, S/P BMT |

Note:

Normal reference values: Lymphocytes 4000-12000 per mm^3^, CD3 3100-4800 per mm^3^, CD4 2200-3300 per mm^3^, CD8 1100-1700 per mm^3^, CD19 1100-1900 per mm^3^, CD16^+^56^+^ 300-700 per mm^3^, IgG 2.5-9.1 g/L, IgA 0.2-1.2 g/L, IgM 0.2-1.5 g/L, IgE 1.6-30 g/L and PHA 94935-171149 CPM.

FTT: failure to thrive

s/p BMT: status post Bone Marrow Transplantation

CMV: cytomegalovirus

ND: not done

Mat Cell: maternal cells engraftment

Mos: months
