# Supplementary material for: Molecular analysis of T-B-NK+ severe combined immunodeficiency and Omenn syndrome cases in Saudi Arabia
Source: BMC Med Genet. 2009 Nov 13;10:116. doi: 10.1186/1471-2350-10-116 (PMC2780402; doi:10.1186/1471-2350-10-116)
Supplement: Additional file 3 — Table S3 - Mutations detected in patients with T-B-NK+ Severe combined immunodeficiency. Mutations listing. [file 1471-2350-10-116-S3.doc]

**Table 3- Mutations detected in patients with T^-^B^-^NK^+^ Severe combined immunodeficiency**

| Patients | Gene | Nucleotide | Amino Acid | Mutation Type | Novel mutation? | Domain  affected | Outcome |
| --- | --- | --- | --- | --- | --- | --- | --- |
| 1 | RAG1 | G1297A | V433M | missense | NO | Nonamer Binding Domain (NBD) | Similar physico-chemical property. Both residues are medium size and hydrophobic |
| 2 | RAG1 | G1297A | V433M | missense | NO | Nonamer Binding Domain (NBD) | Similar physico-chemical property. Both residues are medium size and hydrophobic |
| 3 | RAG1 | G1871A | R624H | missense | NO | Catalytic Core | Change from large size and basic (R) to medium size and polar (H) |
| 4 | RAG1 | C1179T | R394W | missense | NO | Nonamer Binding Domain (NBD) | Change from large size and basic (R) to large size and aromatic (W) |
| 5 | RAG1 | G1677T | R559S | missense | NO | Catalytic Core | Change from large size and basic (R) to small size and polar (S) |
| 6 | RAG1 | G1677T | R559S | missense | NO | Catalytic Core | Change from large size and basic (R) to small size and polar (S) |
| 7 | RAG2 | A379T | K127X | nonsense | YES | Catalytic Core | Truncated RAG2, lacking the nuclear localization signal (NLS) |
| 8 | RAG2 | A379T | K127X | nonsense | YES | Catalytic Core | Truncated RAG2, lacking the nuclear localization signal (NLS) |
| 9 | RAG2 | 49ins13 | S18X | nonsense | YES | Catalytic Core | Truncated RAG2, lacking the nuclear localization signal (NLS) |
| 10 | RAG2 | C10T | Q4X | nonsense | YES | All domains | Truncated RAG2, lacking essentially all domains |
| 11-15 | *DCLRE1C* | del exons 1-3 | - | Gross deletion | YES | All or minimum the N-terminus | Truncated or complete absence of the encoded DCLRE1C protein product |
| 16-21 | Wild Type *RAG1/2&DCLRE1C* | - | - | - | - | - | - |
| 22 | *DCLRE1C* | del exons 1-3 | - | Gross deletion | YES | All or minimum the N-terminus | Truncated or complete absence of the encoded DCLRE1C protein product |
