# Supplementary material for: Molecular analysis of T-B-NK+ severe combined immunodeficiency and Omenn syndrome cases in Saudi Arabia
Source: BMC Med Genet. 2009 Nov 13;10:116. doi: 10.1186/1471-2350-10-116 (PMC2780402; doi:10.1186/1471-2350-10-116)
Supplement: Additional file 4 — Table S4 - Mutations detected in patients with Omenn syndrome. Mutations listing. [file 1471-2350-10-116-S4.doc]

**Table 4- Mutations detected in patients with Omenn syndrome**

| Patients | Gene | Nucleotide | Amino Acid | Mutation Type | Novel | Domain | Outcome |
| --- | --- | --- | --- | --- | --- | --- | --- |
| OS1 | *RAG1* | T1201C | S401P | missense | NO | Nonamer Binding Domain (NBD) | Change from small size and polar (S) to medium size and hydrophobic (P) |
| OS2 | *RAG1* | T1201C | S401P | missense | NO | Nonamer Binding Domain (NBD) | Change from small size and polar (S) to medium size and hydrophobic (P) |
| OS3 | *-* | - | - | - | - | - | - |
| OS4 | *RAG1* | G1187A | R396H | missense | NO | Nonamer Binding Domain (NBD) | Change from large size and basic (R) to medium size and polar (H) |
| OS5 | *RAG1* | G1187A | R396H | missense | NO | Nonamer Binding Domain (NBD) | Change from large size and basic (R) to medium size and polar (H) |
| OS6 | *RAG2* | C1332G | I444M | missense | YES | Catalytic Core | Similar physico-chemical property. Both residues are medium size and hydrophobic |
| OS7 | *DCLRE1C* | del exons  1-3 | - | Gross deletion | YES | All or minimum the N-terminus | Truncated or complete absence of the encoded DCLRE1C protein product |
